# Real-world eligibility for FSGS clinical trials: insights from a US health system

**DOI:** 10.1093/ckj/sfaf377

**Published:** 2025-12-03

**Authors:** Mercedes A Munis, Qiaoling Chen, Alisha Smith, Candelaria L Garcia, David Fuller, John J Sim

**Affiliations:** Department of Research & Evaluation, Kaiser Permanente Southern California, Pasadena, CA, USA; Department of Research & Evaluation, Kaiser Permanente Southern California, Pasadena, CA, USA; Dimerix Bioscience Pty Ltd, Melbourne, VIC, Australia; Department of Research & Evaluation, Kaiser Permanente Southern California, Pasadena, CA, USA; Dimerix Bioscience Pty Ltd, Melbourne, VIC, Australia; Department of Research & Evaluation, Kaiser Permanente Southern California, Pasadena, CA, USA; Division of Nephrology and Hypertension, Kaiser Permanente Los Angeles Medical Center, Los Angeles, CA, USA; Department of Clinical Science, Kaiser Permanente Bernard J. Tyson School of Medicine, Pasadena, CA, USA

To the Editor,

Focal segmental glomerulosclerosis (FSGS) is a heterogeneous podocytopathy with diverse etiologies and clinical courses, often marked by frequent relapses and variable treatment responses. While all forms share characteristic segmental lesions on kidney biopsy, classification into primary, genetic, and secondary types [1] does not fully capture clinical distinctions. Clinical features such as the presence of nephrotic syndrome and the absence of systemic chronic conditions are commonly used to distinguish primary FSGS from secondary forms. Among adults with biopsy-confirmed glomerular disease, FSGS accounts for an estimated 20%–40% of cases and represents a leading pathway to end-stage kidney disease (ESKD) [1, 2, 3]. With no formally approved treatments there is an urgent need for therapies that offer durable efficacy and favorable safety profiles.

Due to substantial intra- and inter-patient variability in eGFR among individuals with FSGS, findings from the Proteinuria and GFR as Clinical Trial Endpoints in Focal Segmental Glomerulosclerosis (PARASOL) and United Kingdom National Registry of Rare Kidney Diseases (RaDaR) studies have suggested that the sample sizes required for clinical trials using eGFR or eGFR slope as endpoints are prohibitively large for most anticipated treatment effect sizes [4]. As therapeutic development efforts continue to expand, characterizing trial eligibility in real-world patient populations is necessary to inform study design and improve applicability to routine clinical practice.

To better understand the real-world landscape of trial eligibility, we conducted a cross-sectional study within Kaiser Permanente Southern California from 2018 to 2024. We identified patients aged 12–80 years with a kidney biopsy demonstrating FSGS as the primary diagnosis. Patient demographic and clinical characteristics were extracted from administrative data sources and electronic health records within 1-year before (baseline period). Using the most recent outpatient laboratory results, we applied inclusion and exclusion criteria reflective of A Study of the Efficacy and Safety of DMX-200 in Patients With FSGS Who Are Receiving an ARB (ACTION3) (https://clinicaltrials.gov/study/NCT05183646). These included an estimated glomerular filtration rate (eGFR) between 25 and 120 ml/min/1.73 m² and urine protein-to-creatinine ratio (UPCR) >1.5 g/g. We excluded biopsies related to transplant, nephrectomy, or reported as secondary FSGS. No exclusions were applied for obesity-related adaptive podocytopathy, genetic variants, or autoimmune markers, reflecting real-world limitations of conventional definitions. Descriptive statistics were used for baseline demographics and clinical characteristics of patients potentially eligible for the ACTION3 study.

Among 3142 biopsy-confirmed FSGS patients, 926 (29.5%) had primary, genetic, or undetermined FSGS; only 173 (18.7%) met trial eligibility criteria. Patients were excluded where FSGS was not the principal diagnosis on kidney biopsy (*n* = 1773; 56.4%) or if age was outside the 12–80-year range (*n* = 128; 0.4%). A total of 351 FSGS patients with UPCR <1.5 and 104 patients with eGFR ≥25 and ≤120 ml/min/1.73 m² were excluded (Fig. 1). Among the trial-eligible cohort, the mean age was 52.5 years (SD 16.9), with 56.6% male. The cohort was racially and ethnically diverse: 40.5% Hispanic, 20.8% White, 24.3% Asian/Pacific Islander, and 12.1% Black. The median eGFR was 44.3 ml/min/1.73 m² (IQR 33.3–66.6), and median UPCR was 2.9 g/g (IQR 2.1–4.1). Severe obesity, defined as a body mass index (BMI) of 40 kg/m² or greater, was present in 11% of the cohort. Among trial-eligible patients with available medication data (*n* = 133), 39.3% were prescribed angiotensin receptor blockers, 25.4% received ACE inhibitors, and 21.4% were treated with immunosuppressive agents within the past year (Supplemental Table 1).

**Figure 1:**
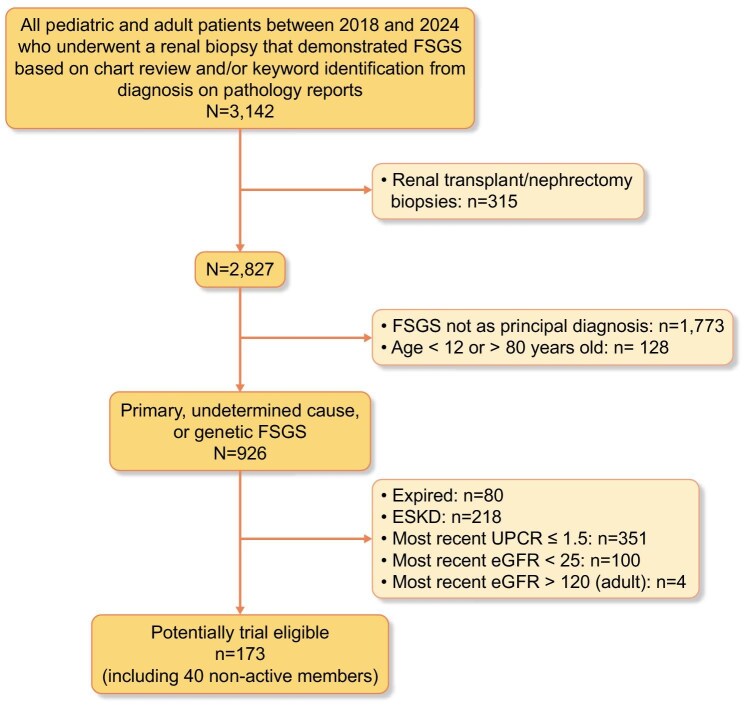
Consort diagram of eligible patients with FSGS.

Trial-eligible FSGS patients comprise a small fraction of the real-world population, underscoring recruitment challenges while there is an urgent need for therapies with durable efficacy. FSGS treatments often fail to achieve lasting remission. Nearly half of patients never reach complete remission, and many relapse or have persistent proteinuria despite therapy. A recent retrospective adult FSGS cohort study from the USA observed that almost half of FSGS patients achieved remission with immunosuppression, however, relapse rates were nearly 70% at 2 years [5]. Given the poor clinical outcomes among patients with this rare kidney disease characterized by remission, relapse, and progression to kidney failure, novel therapies that are efficacious with durable benefit and favorable safety profiles are needed. Our findings illustrate the challenges in identifying and recruiting patients for therapeutic studies in FSGS, as individuals are often captured at different phases in their disease course. This retrospective study is limited by the availability and accuracy of existing records. Furthermore, our analysis was conducted within a single, integrated healthcare system, and therefore the data may not be generalizable to other settings. In addition, the conventional concept of FSGS diagnosis applied by our study based on biopsy and proteinuria thresholds is evolving. Our approach did not exclude patients with severe obesity who may have adaptive podocytopathy, potentially inflating estimates of the primary FSGS population. Furthermore, genetic forms of podocytopathy were not rigorously identified due to the absence of systematic genetic screening. Autoimmune podocytopathies could not be distinguished without serum autoantibody testing that was available. These limitations underscore the need for modernized eligibility frameworks in FSGS trials. Future studies should incorporate genetic testing, standardize criteria to exclude adaptive podocytopathy (e.g. BMI-based thresholds), and immunologic markers (as they become available) or remission/relapse patterns to better capture autoimmune forms. These refinements will help trial cohorts lessen heterogeneity and improve the interpretation of treatment effects.

## Supplementary Material

sfaf377_Supplemental_File
