# Supplementary material for: Real-world eligibility for FSGS clinical trials: insights from a US health system
Source: Clin Kidney J. 2025 Dec 3;19(2):sfaf377. doi: 10.1093/ckj/sfaf377 (PMC12877409; doi:10.1093/ckj/sfaf377)
Supplement: sfaf377_Supplemental_File [file sfaf377_supplemental_file.docx]

**Supplemental File**

**References**

**(1-5)**

1. Mariani LH, Trachtman H, Thompson A, et al.; Proteinuria as an End Point in Clinical Trials of Focal Segmental Glomerulosclerosis. Am J Kidney Dis 2025; 85(5):610-617.

2. De Vriese AS, Sethi S, Nath KA, Glassock RJ, Fervenza F; Differentiating primary, genetic, and secondary FSGS in adults: a clinicopathologic approach. J Am Soc Nephrol 2018; 29(3):759-774.

3. A pivotal phase 3, multicenter, randomized, double-blind, placebo-controlled study of the efficacy and safety of DMX-200 in patients with focal segmental glomerulosclerosis (FSGS) who are receiving an angiotensin II receptor blocker (ARB). ClinicalTrials.gov 2021.

4. Drexler Y, Athavale A, Smith AR, et al.; Clinical Outcomes in the Nephrotic Syndrome Study Network: Disease Burden and Treatment Patterns over Time. Glomerular Dis 2025; 5(1):288-303.

5. Pitcher D, Braddon F, Hendry B, et al.; Long-Term Outcomes in Nephrotic Syndrome by Kidney Biopsy Diagnosis and Proteinuria. Journal of the American Society of Nephrology 2025; 36(7):1398-1413.

**Supplemental Table 1. Patient characteristics of potentially trial eligible patients.**

|  | Total (N=173) |
| --- | --- |
| Age in years as of 04-25-2025 |  |
| Mean (SD) | 52.5 (16.9) |
| Median (IQR) | 54.3 (38.4, 67.8) |
| Gender, n (%) |  |
| Female | 75 (43.4%) |
| Male | 98 (56.6%) |
| Race/ethnicity, n (%) |  |
| Asian/Pacific Islander | 42 (24.3%) |
| Black | 21 (12.1%) |
| Hispanic | 70 (40.5%) |
| White | 36 (20.8%) |
| Other/unknown | 4 (2.3%) |
| Most recent outpatient eGFR^1^ |  |
| Mean (SD) | 53.9 (25.6) |
| Median (IQR) | 44.3 (33.3, 66.6) |
| Most recent outpatient UPCR |  |
| Mean (SD) | 3.5 (2.0) |
| Median (IQR) | 2.9 (2.1, 4.1) |
| Most recent BMI |  |
| Mean (SD) | 31.3 (7.0) |
| Median (IQR) | 31.2 (25.9, 35.5) |
| Normal weight (BMI 18.5-24.9), n (%) | 36 (20.8%) |
| Overweight (25-29.9), n (%) | 80 (46.2%) |
| Obesity (30-39.9), n (%) | 38 (22.0%) |
| Severe Obesity (40+), n (%) | 19 (11.0%) |
| Elixhauser comorbidity index^2^ |  |
| Mean (SD) | 3.3 (2.8) |
| Median (IQR) | 3.0 (1.0, 5.0) |
| Medication use in 2025^3^, n (%) |  |
| Angiotensin-converting enzyme (ACE) inhibitors | 44 (25.4%) |
| Angiotensin-receptor blockers (ARB) | 68 (39.3%) |
| Dual endothelin and angiotensin II receptor antagonists (DEARA) | 0 |
| Mineralocorticoid receptor antagonists (MRA) | 20 (11.6%) |
| Sodium Glucose Contransporter-2 Inhibitors (SGLT-2i) | 73 (42.2%) |
| Immunosuppressant^4^ | 37 (21.4%) |
| Corticosteroids | 27 (15.6%) |
| *Budesonide* | 1 (0.6%) |
| Alkylating Agents | 1 (0.6%) |
| Calcineurin inhibitors | 10 (5.8%) |
| Other | 10 (5.8%) |

**^1^**Per the 2009 CKD-EPI glomerular filtration rate (GFR) equation. **^2^**Value is based on diagnosis within the past year. **^3^**Prescribed medication data captures active members only. **^4^**Immunosuppressive agents are not mutually exclusive
